# *MiR-216a-3p* inhibits the cytotoxicity of primary natural killer cells

**DOI:** 10.3389/fonc.2024.1523068

**Published:** 2025-01-21

**Authors:** Rowan Abdelbary, Manon Ragheb, Shereen A. El Sobky, Nagwa El-Badri, Nourhan Aboud, Ahmed Tawheed, Asmaa Gomaa, Mona Zidan, Ramy K. Aziz, Abd Elrahman Abouzid, Radwa Ayman Salah, Mohamed El-Kassas, Imam Waked, Ahmed Moustafa, Injie Omar Fawzy, Nada El-Ekiaby, Ahmed Ihab Abdelaziz

**Affiliations:** ^1^ Biotechnology Graduate Program, American University in New Cairo, Cairo, Egypt; ^2^ School of Medicine, Newgiza University (NGU), Giza, Egypt; ^3^ Center of Excellence for Stem Cells and Regenerative Medicine, Zewail City of Science and Technology, Giza, Egypt; ^4^ Endemic Medicine Department, Faculty of Medicine, Helwan University, Cairo, Egypt; ^5^ National Liver Institute, Menoufia University, Menoufia, Egypt; ^6^ Microbiology and Immunology Research Program, Children’s Cancer Hospital Egypt 57357, Cairo, Egypt; ^7^ Department of Biology, American University in Cairo, New Cairo, Egypt

**Keywords:** microRNAs (miRNAs), miR-216a-3p, hepatocellular carcinoma (HCC), natural killer (NK) cells, tumor necrosis factor-alpha (TNF-alpha), interferon-gamma (IFN-gamma), granzymes (GrB), perforins (PRF)

## Abstract

**Introduction:**

The role of miRNAs in regulating variable molecular functions has been sought by scientists for its promising utility in regulating the immune response and, hence, in treating various diseases. In hepatocellular carcinoma (HCC) specifically, a reduction in the number and efficiency of circulating and intrahepatic natural killer (NK) cells has been reported. Our project aims to investigate the role of *miR-216a-3p* in the regulation of NK cell cytotoxicity, especially since it plays a tumor suppressor role in the context of HCC.

**Methods:**

To achieve our aim, we isolated NK cells from the whole blood of 86 patients with HCC and 23 healthy controls. We assessed the expression profile of *miR-216a-3p* in NK cells of patients and controls. Furthermore, we induced the expression of *miR-216a-3p* in NK cells isolated from healthy controls, followed by measuring the release of interferon-gamma (IFN-γ), tumor necrosis factor-alpha (TNF-α), perforins (PRF) and granzyme B (GrB) using ELISA as well as NK cells cytolytic activity against Huh7 cells using lactate dehydrogenase (LDH) cytotoxicity assay. After that, we performed an *in silico* analysis to understand the mechanistic regulation imposed by *miR-216a-3p* on NK cells to study its impact on one of its potential downstream targets.

**Results:**

Our results have indicated that *miR-216a-3p* has higher expression in NK cells of patients with HCC, and simulating this elevated expression pattern via forcing *miR-216a-3p* expression in normal NK cells has negatively impacted the release of TNF- α, IFN- γ, GrB, and PRF. Consequently, a decrease in cell cytolysis was observed. Our *in silico* analysis revealed that the predicted downstream targets of *miR-216a-3p* are enriched in the FOXO-signaling pathway. Among those targets is FOXO-1, which has been reported to play a role in NK cell maturation. Thus, we evaluated FOXO-1 expression upon mimicking *miR-216a-3p* in control NK cells that showed significant downregulation of FOXO-1 on both RNA and protein levels.

**Conclusion:**

In conclusion, we report *miR-216-3p* as a negative regulator of NK cell cytotoxicity.

## 1 Introduction

Cancer cells are well-known to escape immune surveillance, leading to immune evasion and colonization (1). Natural killer (NK) cells have emerged as promising immunotherapeutic tools against cancers (2–4). In fact, the clinical application of NK cells as a therapeutic option for hematological malignancies has mainly demonstrated promising effects (2, 5). However, NK therapy of solid tumors is currently under serious investigation (3, 6–8). NK cells are crucial members of the innate immune response that scan the body for virally infected and tumor cells and directly eradicate them with no need for prior sensitization; hence, they are considered the body’s first line of defense, which is the reason behind their immunotherapeutic potential (4, 9). NK cells induce their cytotoxic activity through a variety of activating and inhibitory receptors (10, 11). The strict balance between the turn-on signals received by the activating receptors and the turn-off signals received by the inhibitory ones ultimately determines the state of NK cell activity (12). In cancers, NK cell cytolytic functions are impaired with frequent imbalances between the activating and inhibitory signals, allowing tumor cells to evade the NK cell-mediated immune responses (13). In HCC specifically, a reduction in the number of circulating and intrahepatic NK cells has been reported (14–16). Moreover, various studies have shown that NK cells from patients with HCC exhibited defective cytotoxicity and cytokine secretion compared to NK cells from healthy donors, which facilitated immune-evasion (16–21). The impairment in NK cells has been correlated to the decrease in the anti-tumor immune response due to a decrease in cytotoxicity and interferon-gamma (IFN-gamma) production, accompanied by an increase in the suppressive Treg cells (14). Yan Wu et al. have shown that NK cell exhaustion and death take place in HCC due to defective monocyte-NK cell activation, marked by a subsequent decrease in tumor necrosis factor-alpha (TNF-α) and IFN-gamma production (16). Jason Chiang et al. have demonstrated that the use of stimulated primary NK cells in HCC was able to activate T-cell activity and induce cytotoxicity (17). We have shown that NK cell cytotoxicity can be regulated by manipulating the expression of microRNAs (miRNAs) (21–24).

MiRNAs have been comprehensively reported to play a key role in the regulation of NK cells, in terms of maturation, development and function (25, 26). Interestingly, miRNAs frequently exhibit opposing roles in different tissue types (27). For example, we have reported the contradicting role of *miR-182*, as it induced the expression of the activating receptor *NKG2D* and enhanced NK cell cytotoxicity, hence supporting tumor eradication, while it has also been shown to act as an oncomiR in HCC cells through the inhibition of *FOXO3a* (24). Moreover, we showed that *miR-615-5p* suppresses *NKG2D* as well as NK cytolytic function (23). Nevertheless, *miR-615-5p* has been shown to act as a tumor suppressor in liver cancer through the inhibition of *IGF-2* and *SHMT2* (28, 29). In light of the varying functions of miRNAs according to the cellular context, dissecting the role of miRNAs in regulating immune cell cytotoxicity has become inevitable.

*MiR-216a* is composed of two isoforms: *miR-216a-5p* and *miR-216a-3p* (30). This miRNA has been researched for its controversial role in the progression of various solid tumors; nevertheless, there is a gap in determining its exact role in HCC and immunity. Much effort has been invested in examining the role of *miR-216a-5p*; it was shown to exploit a tissue-specific vacillating behavior (30), where it was reported to act as a tumor suppressor in many cancers while being an oncomiR in others. In addition, it was also reported as one of the immune-modulating miRNAs in colorectal cancer (30–33). On the other hand, *miR-216a-3p* has seldom been scrutinized. In a study by Wu et al., *miR-216a-3p* was shown to act as an oncomiR in gastric cancer by promoting the NF-kB pathway (34). On the contrary, it showed a tumor suppressor effect acting in colorectal cancer through the negative regulation of *COX2* and *ALOX5* (35). Interestingly, *miR-216a-3p* has been shown to negatively impact the tumor’s immune environment in colon adenocarcinoma, causing an imbalanced immune response and tumor progression (36). The role *of miR-216a-3p* in liver pathology has rarely been investigated except for one study that has shown that *miR-216a-3p* upregulation alleviated the sorafenib resistance in HCC xenografts of nude mice by inhibiting the MAPK14 pathway (37).

These studies taken together highlight the necessity of understanding the controversial roles of miRNAs generally, and of *miR-216a-3p* specifically, in regulating their molecular function, and of assessing the role of these molecules in NK cytotoxicity. Our previous research has shown that NK cell dysfunction in patients with HCC is amenable to correction by epigenetic manipulations, where NK cells of patients with HCC show impaired cytolytic activity against tumor cells (21, 22). Given the promising role of *miR-216a-3p* as an immune modulator in colorectal cancer, our interest was in investigating the role of *miR-216a-3p* in NK cell activity and assessing whether it has an anti-cytotoxic or pro-cytotoxic role.

## 2 Materials and methods

### 2.1 Sample collection

Peripheral venous blood (10 ml) was collected from 23 healthy controls and 86 patients with HCC in the presence of an anticoagulant (EDTA). A larger cohort of patients was required to reach an adequate NK cell count sufficient for screening experiments due to the decreased number of circulating NK cells in patients with HCC compared to controls. All samples were processed on the same day and within a few hours after collection. Written informed consent was obtained from all patients. Patients were recruited from Badr Hospital, Helwan University, and the National Liver Institute, Menoufia University in Egypt. All experiments were conducted under the ethical standards of the Declaration of Helsinki and were approved by the institutional review boards of Helwan University (8-2021) and Menoufia University (00503/2023). Patients underwent clinical assessment and information on age, gender, presence of HCV and HBV, and BCLC staging were recorded (**Supplementary Table 1**).

### 2.2 Ficoll separation

Peripheral blood mononuclear cells (PBMCs) were isolated from blood samples using the Ficoll density gradient centrifugation method as previously described (21–23). Fresh venous blood was diluted with an equal volume of wash mix (RPMI, Penicillin/Streptomycin 1% and FBS 5%). Diluted blood was layered on the walls of tubes containing Ficoll hydrate (HiSep™ LSM 1077, HIMEDIA). The PBMC layer was collected after centrifugation at 1000 rpm for 30 minutes and washed three consecutive times to remove platelets. PBMCs were resuspended in 1ml freeze mix (RPMI, 30% FBS, 10% DMSO) (LONZA, Belgium) and stored at -80°C for later use.

### 2.3 NK cells isolation and purity assessment

Natural killer cells were isolated from pooled PBMCs using the EasySep human NK cell isolation kit (Stem Cell Technologies) according to the manufacturer’s instructions, which follows a magnetic-based negative selection process (EasySep™ Magnet, Stem Cell Technologies). The purity of NK cells were determined using flow cytometric analysis of CD56 and CD3 surface markers, as described in previous studies (22, 23, 38). A CytoFLEX V5-B5-R3 flow cytometer (13 detectors, three lasers) was used for the assessment of NK cell purity. Isolated NK cells with at least 90% purity were utilized for all further expression profiling and functional analysis experiments.

### 2.4 Cell culture

NK cells were cultured at 37°C and 5% CO_2_ in full RPMI (Lonza, Belgium) supplemented with 5% fetal bovine serum (FBS; Lonza, Belgium) and 1% Penicillin/Streptomycin (BioWhittaker, Lonza, Belgium), as previously described (22, 23). Cells were supplemented with 100 IU of IL-2 on day 1 and 500 IU of IL-2 on day 2 for maintenance and activation (Recombinant Human IL-2 202-IL2-010, R&D Biotech) (39).

Huh-7 cells were cultured at 37°C and 5% CO_2_ in full DMEM (Lonza, Belgium) supplemented with 5% FBS and 1% Penicillin/Streptomycin. Cells were split upon reaching 80% confluency.

### 2.5 Transfection

NK cells cultured in a 96-well plated at a density of 75 × 10^3^ cells/well were transfected with 100nM of either *miR-216a-3p* (identified by the manufacturer as mimics) or Allstars negative control oligonucleotides (1027292, Qiagen, Germany) using lipofectamine 3000 transfection reagent (L3000001, Invitrogen, USA) according to the manufacturer’s instructions. Three biological repeats were performed with three technical replicates each.

### 2.6 RNA extraction

Total RNA was isolated from NK cells using the miRNeasy column extraction method with the advised DNase step, according to the manufacturer’s instructions (217084, Qiagen, Germany).

### 2.7 Reverse transcription and real-time quantitative PCR

Extracted RNA was reverse transcribed into complementary DNA (cDNA) using high-capacity (4368814, Invitrogen, USA) and miScript reverse transcription kits (218160, Qiagen, Germany). The genes of interest were amplified using SYBR Green-based quantitative real-time-polymerase chain reaction (qRT-PCR) on a QuantStudio Real-Time PCR instrument (Applied Biosystems, USA) and software (Applied Biosystems), as per the manufacturer’s instructions. RT-qPCR was used to assess the expression of *miR-216a-3p*, RNU6B, RPL41 and FOXO-1, where RNU6B was used for the normalization of *miR-216a-3p* expression, while RLP41 was used as a housekeeping gene for the normalization of FOXO-1 expression.

### 2.8 Flow cytometry

For flow cytometry analysis, negative control oligonucleotides- and *miR-216a-3p*-transfected primary NK cells were centrifuged, and the cell supernatant was discarded. Cells were then resuspended in 4% formaldehyde and fixed at room temperature (RT) for 15 minutes. Then, cells were washed by centrifugation and resuspended in 1 ml PBS. Cells were permeabilized using ice-cold methanol (100%) to a final concentration of 90% methanol and left to permeabilize for 10 minutes on ice. Immunostaining was then performed via washing cells by centrifugation in excess PBS and resuspended in diluted FOXO-1 primary antibody (Cell Signaling Technology, USA) prepared in antibody dilution buffer and incubated for 1 hour. Cells were then washed by centrifugation in antibody dilution buffer, and the supernatant was discarded. Cells were resuspended in 100 µl of diluted Alexa Fluor^®^555 anti-rabbit IgG secondary antibody (Cell Signaling Technology, USA) and incubated for 30 minutes at RT, protected from light. Washing by centrifugation was then performed in antibody dilution buffer, and the supernatant was discarded. Cells were then resuspended in 300 µl PBS and 2,000 cells per sample were analyzed on a FACSCalibur™ (Becton Dickinson, USA) following standard operating procedures and analyzed using CellQuest Pro Software (Becton Dickinson, USA) as described previously (40). Unstained samples were used as a negative control for gating. Data analyses were done utilizing FlowJo v. 10.2 software (Treestar, USA). Two biological experiments were performed with each sample performed in triplicates.

### 2.9 Enzyme-linked immunosorbent assay

Cytokines released in the cell culture supernatant, specifically TNF-α (Human TNF-alpha DuoSet ELISA DY210-05, R&D Biotech) and IFN-gamma (Human IFN-gamma DuoSet ELISA DY285B-05, R&D Biotech), were measured using cytokine-specific ELISA kits. The release of granzyme B (GrB) and perforins (PRF) was assessed using the Human granzymes B (Gzms-B) (YLA0952HU, YLbiont) ELISA kit and the Human Perforin/Pore-forming (PFF/PFP) ELISA Kit (YLA1511HU, YLbiont), respectively, according to the manufacturer’s instruction. The plates were coated on day 1 with the capture (primary) antibody (specific to the target). On day 2, a series of washes were done, followed by blocking, sample addition and detection through the secondary antibody conjugated to streptavidin-HRP. Absorbance was measured at 450 nm. Two biological experiments were performed with three technical repeats for TNF-α and IFN-gamma, while three biologicals were performed for GrB and PRF.

### 2.10 Lactate dehydrogenase assay

After 48 hours of transfection with *miR-216a-3p* mimics, NK cells (10 × 10^4^ cells/well) were co-cultured with target human hepatocellular carcinoma cells (Huh-7) (25 × 10^3^ cells/well in a 96-well plate) with effector-to-target ratio of 4:1, at 37°C and 5% CO_2_ for 24 hours. The extent of lactate dehydrogenase (LDH) release was used to measure the degree of cell damage after the co-culturing of NK cells with target hepatocytes. LDH release was measured using the Lactate Dehydrogenase Activity Assay kit (MAK066-1K1-Sigma-Aldrich) according to the manufacturer’s instructions. After 24 hours of co-culturing, plates were centrifuged, and supernatants were collected and transferred to new plates. CytoTox96 reagent was then added to each well and the plate was covered with foil and incubated for 30 minutes at RT. A stop solution was then added, and any bubbles were removed using a needle. Measurements were taken at 490 nm within 1 hour of adding the stop solution. According to the manufacturer’s instructions, the kit’s lysis solution was used to induce complete lysis of target cells that were not co-cultured with NKs, which served as Target Cell Maximum LDH release control (positive control). Two biological experiments were performed with three technical repeats.

### 2.11 *In silico* prediction

MiRDB, a publicly available target prediction tool, was used for the prediction of potential *miR-216a-3p* target genes (41). The species was set to “Humans”, and searches were performed using the miRNA name. Then, KEGG enrichment analysis was performed with the miR-216a-3p predicted targets through the publicly available ncPath platform (an enrichment analysis and data visualization tool) to determine the enriched pathways. Enriched pathways were manually researched to select those indicated to have a role in NK cell maturation and activity, and HCC tumorigenesis. Members of the selected signaling pathway were cross-checked in the list of targets extracted from the MiRDB tool to predict if they are possible targets of *miR-216a-3p*.

### 2.12 Statistical analysis

Statistical analysis was performed using GraphPad Prism 10.0 Software (GraphPad Software, Inc.). Data are presented as the mean ± standard error of the mean (SEM). Unpaired student t-tests were performed for statistical significance of numeric data. *P*-values of less than 0.05 were considered as an indication of statistical significance.

## 3 Results

### 3.1 Expression profiling of *miR-216a-3p* in natural killer cells of patients with HCC vs. healthy controls

**Figure 1:**
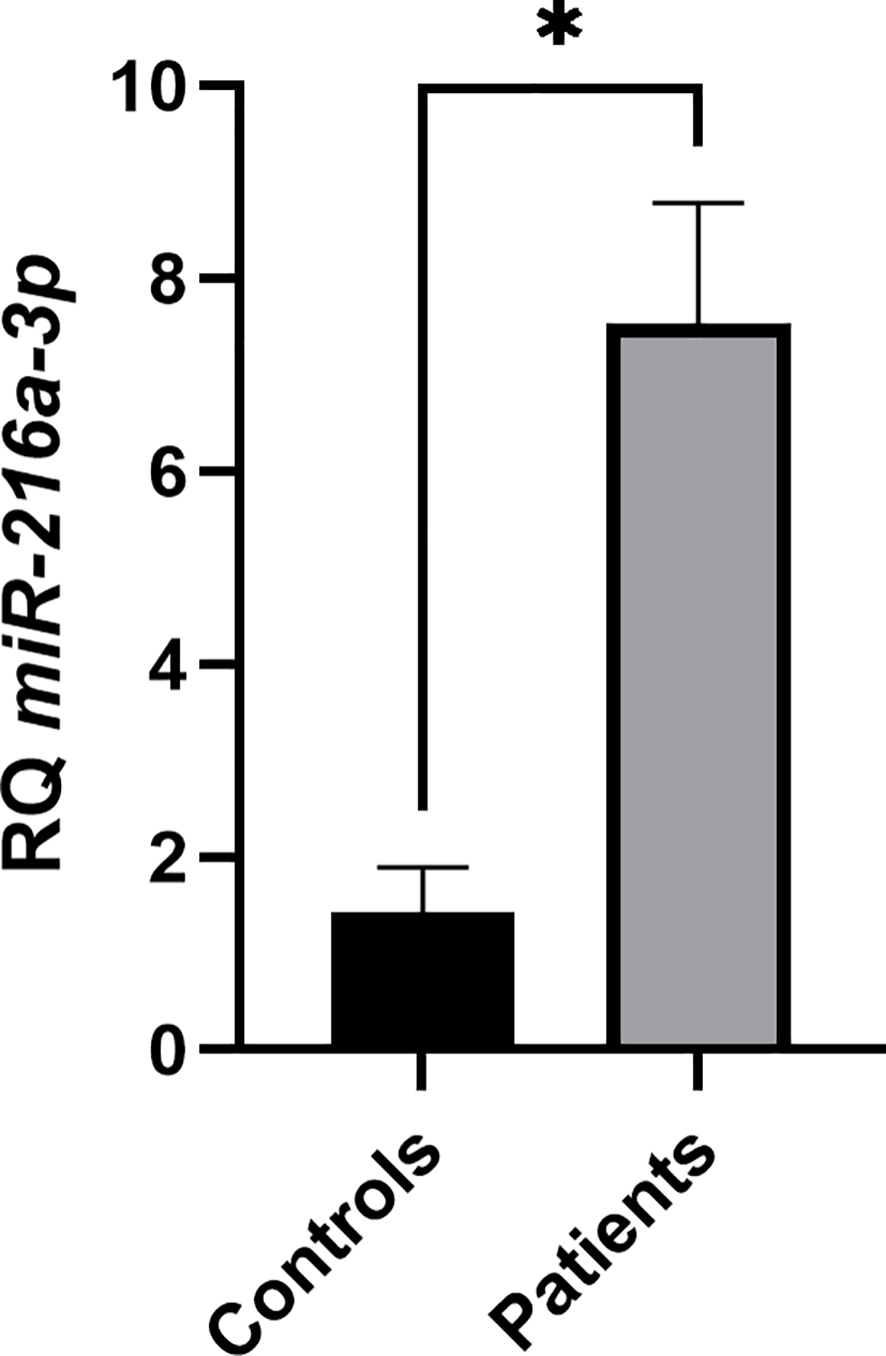
Screening of *miR-216a-3p* in NK cells of patients vs. controls. Expression pattern of *miR-216a-3p* in NK cells isolated from pools of patients with HCC and healthy controls. Results are expressed as mean +/- SEM. Asterisks indicate statistically significant difference, where *p<0.05.

The lack of reports regarding the role of *miR-216a-3p* in NK cells of patients with HCC intrigued us to study the function of *miR-216a-3p* in NK cells and whether it has a negative (anti-cytotoxic) or positive (pro-cytotoxic) role on these cells. However, initially it was informative to determine the expression status of this miRNA in NK cells of patients with HCC compared to controls as a starting point. Thus, the relative expression (RQ) of *miR-216a-3p* was assessed in NK cells isolated from whole blood withdrawn from patients with HCC and healthy controls using RT-qPCR. *miR-216a-3p* was upregulated in NK cells of patients with HCC compared to healthy controls, with a mean relative expression of 7.534 and 1.425 (*p*-value=0.0134), respectively. These results have shown that the mean expression of *miR-216a-3p* in NK cells was higher in patients with HCC compared to healthy controls (**Figure 1**). Thus, we decided to manipulate the levels of *miR-216a-3p* using mimics in primary NK cells of controls to induce an ectopic expression similar to that observed in pools of patients with HCC to determine it functional role.

### 3.2 Impact of *miR-216a-3p* ectopic expression on cytokines and effector molecules release

**Figure 2:**
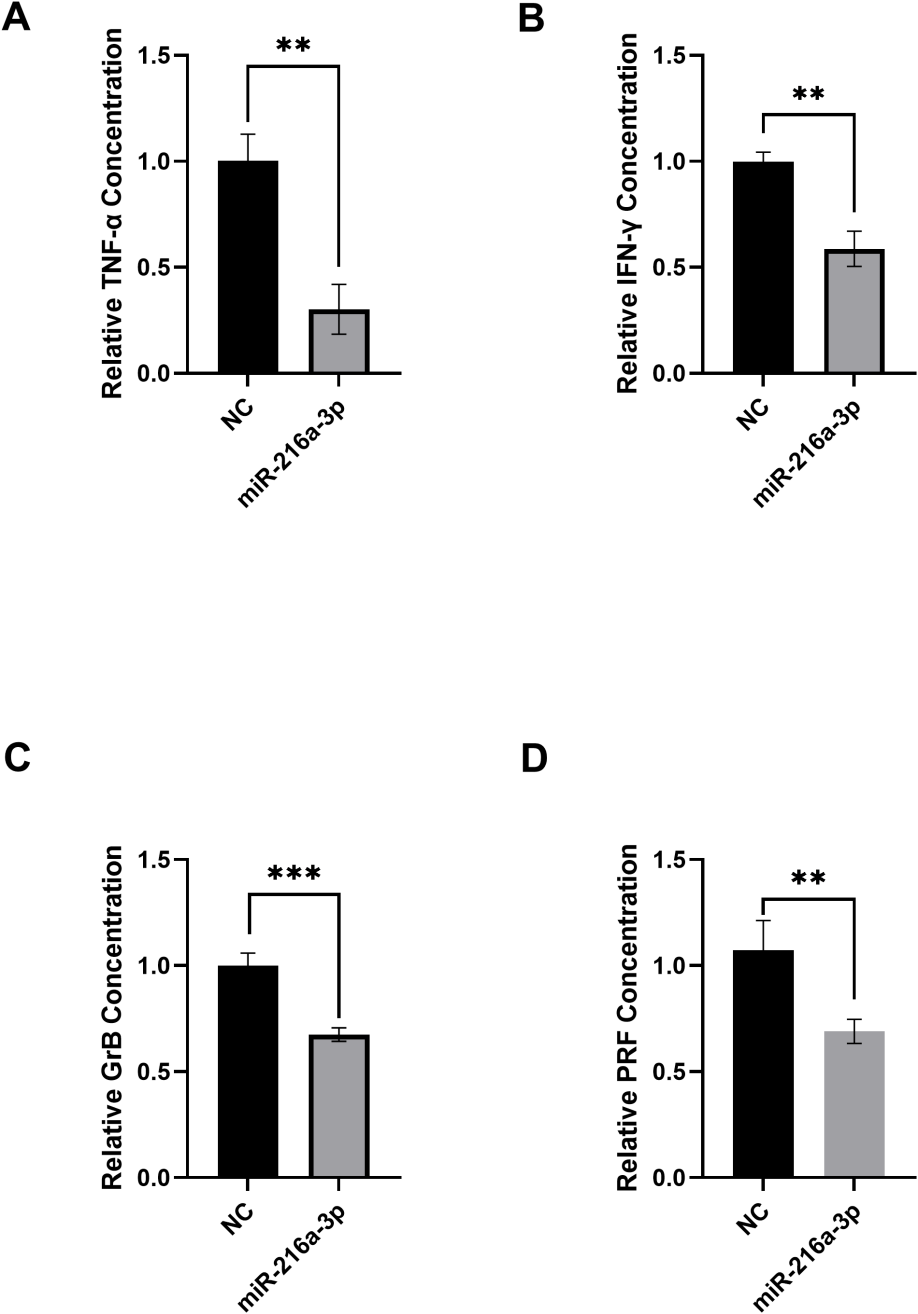
Impact of *miR-216a-3p* mimicking in NKs on TNF-α levels, IFN-gamma release, granzyme B and perforins. **(A)** TNF-α (n=2), **(B)** IFN-gamma (n=2), **(C)** Granzyme B (n=3), and **(D)** Perforins (n=3) levels were assessed using ELISA upon transfecting NK cells with miR-216a-3p mimics. Results are expressed as mean +/- SEM. Asterisks indicate a statistically significant difference, where **p<0.01 and ***p<0.001.

After determining the expression status of *miR-216a-3p* in NK cells of patients with HCC, we proceeded to determine the role of *miR-216a-3p* in NK cell function. We decided to imitate the status of *miR-216a-3p* in the NK cells of patients to assess the impact of this manipulation on the activity of NK cells from healthy controls. We achieved this by transfecting the NK cells isolated from healthy controls with *miR-216a-3p* mimics, followed by measuring the secreted cytokines and cytolytic molecules, TNF-α, IFN-gamma, GrB, and PRF, using ELISA assay. The secretion of TNF-α (n=2, 0.302, *p*-value=0.0024, **Figure 2A**), IFN-gamma (n=2, 0.587, *p*-value=0.0015, **Figure 2B**), GrB (n=3, 0.675, *p*-value=0.0007, **Figure 2C**) and PRF (n=3, 0.690, *p*-value=0.0001, **Figure 2D**) were suppressed upon transfection of *miR-216a-3p* mimics compared to negative control. Hence, *miR-216a-3p* ectopic expression suppressed the release of cytokines and effector molecules from NK cells, represented by the decline in TNF-α, IFN-gamma, GrB, and PRF levels. GrB and PRF production is known to promote the cytolytic activity of NK cells against tumor cells. Thus, we were subsequently interested in studying the impact of *miR-216a-3p* on the cytolytic activity of NK cells against Huh-7 tumor cells.

### 3.3 Impact of *miR-216a-3p* on the cytolytic activity of natural killer cells

**Figure 3:**
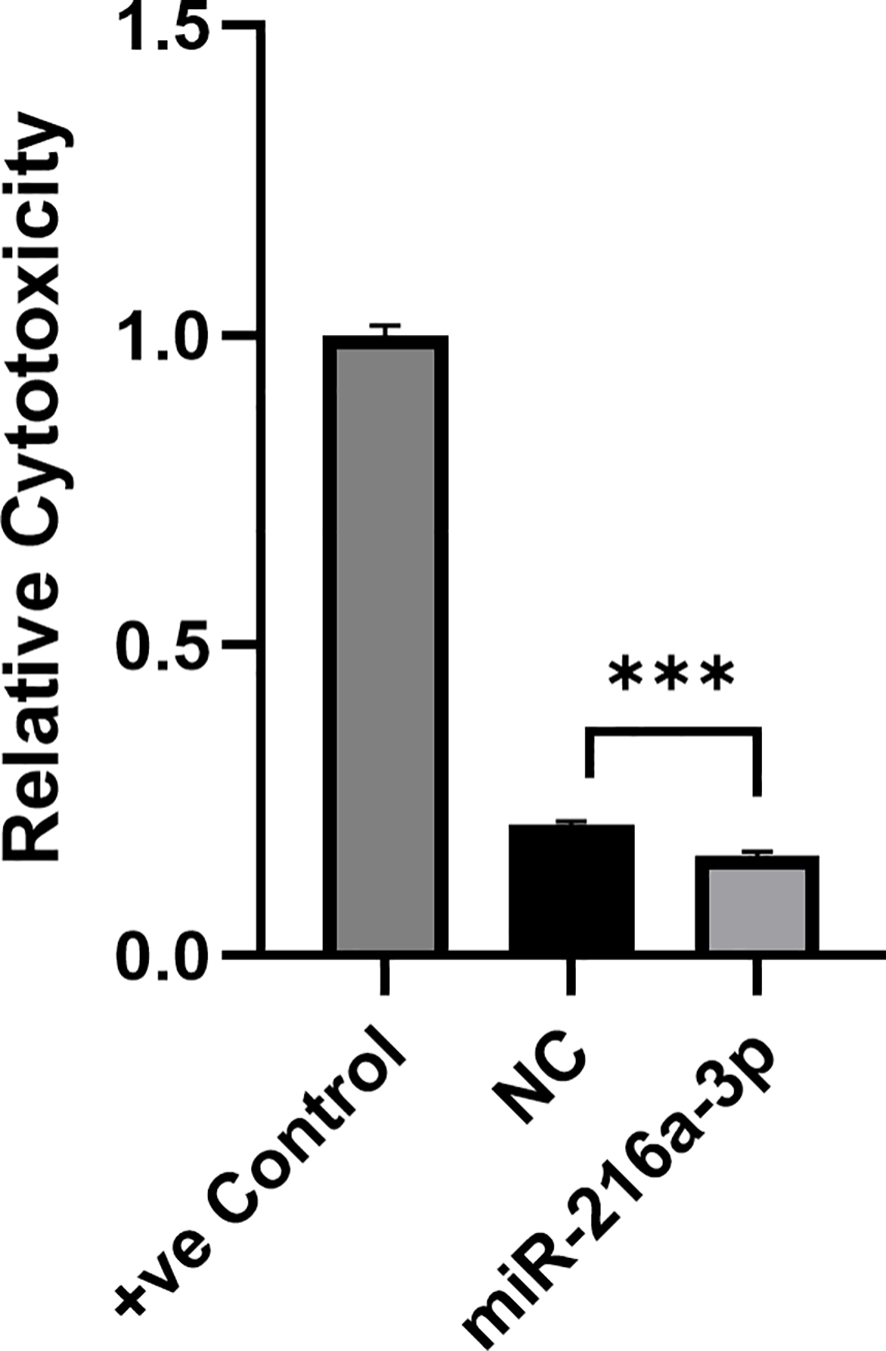
Impact of *miR-216a-3p* mimics in NK cell cytotoxicity through LDH. The cytolytic activity of NK cells against target cells was determined upon NK cell transfection with *miR-216a-3p* mimics and co-culturing with Huh-7 cells, through LDH assay. Results are expressed as mean +/- SEM. Asterisks indicate statistically significant difference, where ***p<0.001, n=2.

We further assessed whether the suppressive effect that *miR-216a-3p* has on the secreted cytokines and cytolytic molecules affects the overall cytotoxic activity of NK cells. Hence, NK cells (effector cells) were transfected with *miR-216a-3p* mimics for 48 hours and were then cocultured with Huh-7 (target cells) at an effector-to-target ratio of 4:1. The cytolytic impact of NK cells on their target Huh-7 cells was assessed using LDH assay (**Figure 3**). The positive control (+ve Control) shows the maximum cytotoxicity (**Figure 3**). A reduction in LDH release from Huh7-cells cocultured with miR-216a-3p transfected NK cells compared to positive control cells was observed, which indicates a significant decrease in NK cell cytolytic activity upon mimicking with *miR-216a-3p* (n=2, 0.161, *p*-value=0.0004). These results along with our previous findings indicate that ectopic expression of *miR-216a-3p* in NK cells causes a decrease in the release of cytokines and effector molecules, as well as a reduction in NK cytolytic activity. Therefore, we were interested in further understanding how *miR-216a-3p* might be imposing its function. Hence, *in silico* analyses were performed as an effort to determine potential NK cell-related targets of *miR-216a-3p*.

### 3.4 Potential downstream target of *miR-216a-3p* regulating natural killer cells activity

**Figure 4:**
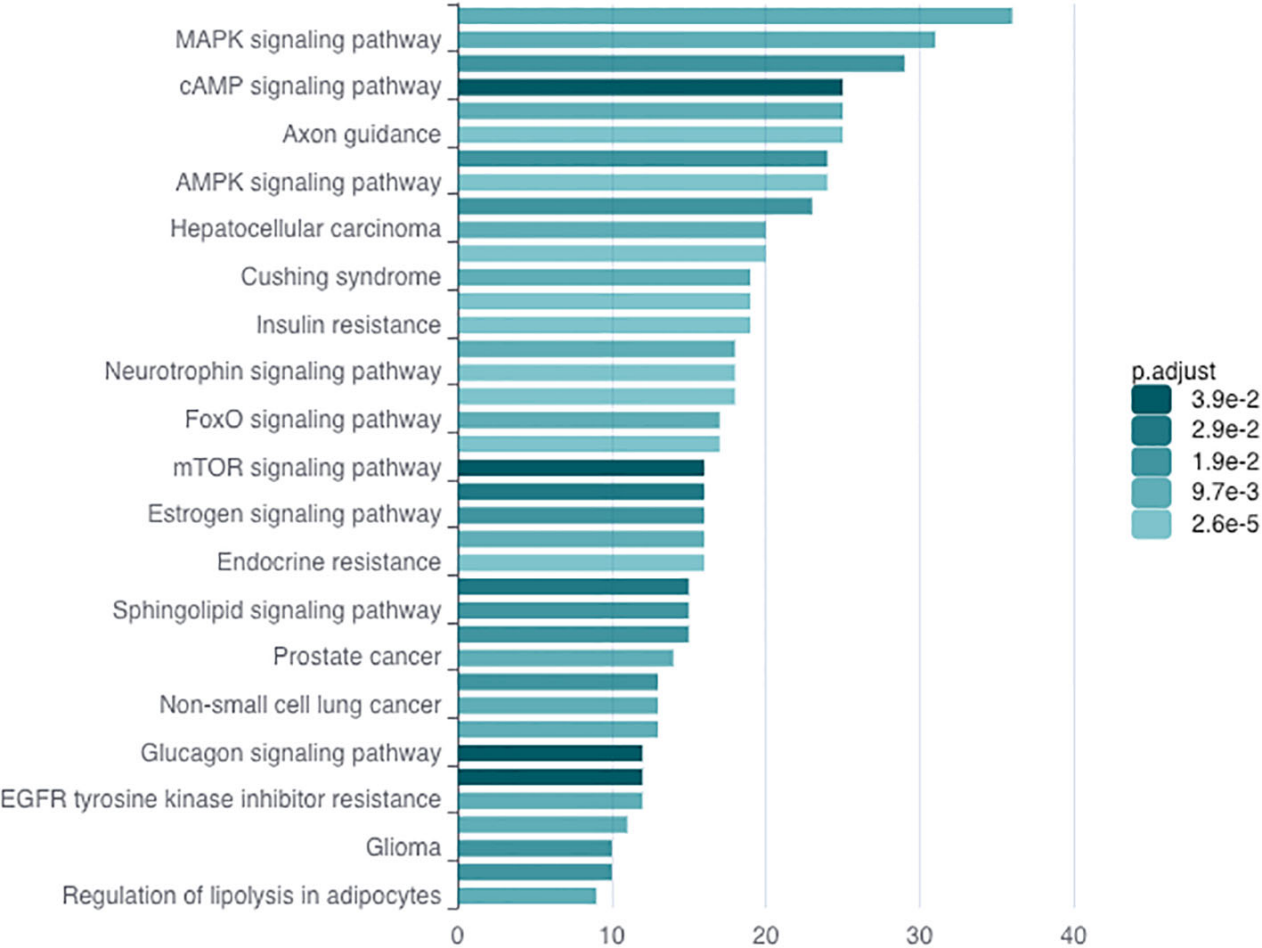
KEGG Enrichment Analysis. KEGG enrichment analysis through ncPath to identify the enriched pathways, among which FoxO signaling pathway was present.

In order to understand how *miR-216a-3p* might be imposing its effects in NK cells, we explored the potential target genes of this miRNA. To determine *miR-216a-3p* targets, an *in silico* analysis was performed using miRDB. Then, we performed KEGG enrichment analysis for the entire list predicted *miR-216a-3p* targets using ncPath to identify the enriched pathways (**Figure 4**) (42). The identified enriched pathways were then manually researched in literature to identify pathways and targets with dual roles in HCC tumorigenesis and NK cell activity regulation. This revealed the MAPK signaling and FoxO signaling pathways as potential candidates for further analysis. Since MAPK signaling pathway has been extensively studied, we focused on the FoxO signaling pathway due to the limited literature regarding its role in NK cells. FoxO signaling pathway was significantly enriched with an adjusted *p*-value of 0.01. *FOXO-1* and *FOXO-4* were the only FoxO family members identified through our bioinformatic searches retrieved from miRDB to be potential *miR-216a-3p* target genes. A literature review was performed for both members, and their protein abundance was checked through the *Human Protein Atlas (Version: 24.0)* which showed a nonspecific and very low expression of FOXO-4 in NK cells and other immune cells, as opposed to FOXO-1. This led to the selection of our candidate, FOXO-1, which has been shown to be a key transcription factor in normal liver, HCC, and NK cell activity (43–45).

**Figure 5:**
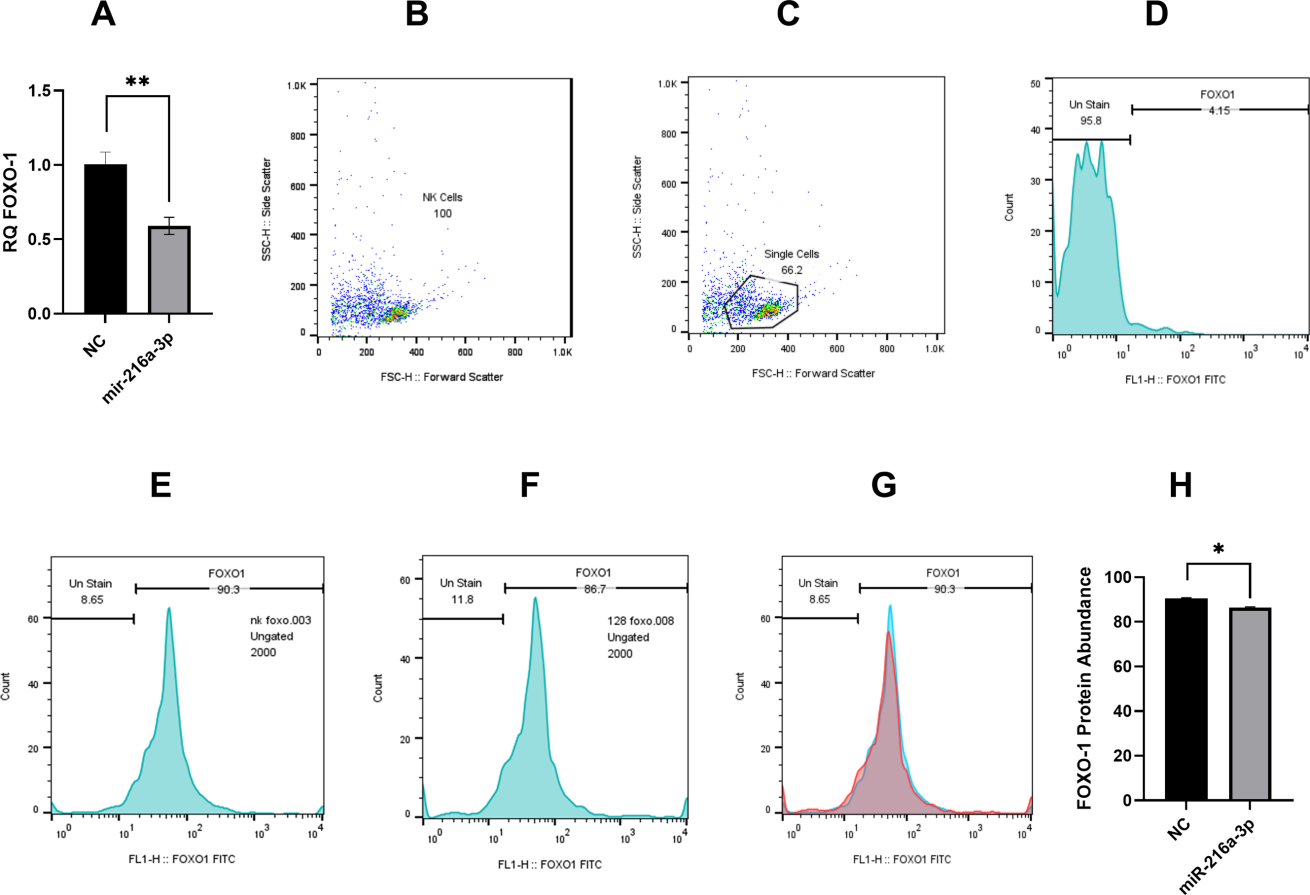
Impact of *miR-216a-3p* on FOXO-1 mRNA and protein expression in NK cells. **(A)** FOXO-1 mRNA level was assessed in response to the introduction of *miR-216a-3p* mimics in NK cells (n=3). **(B–H)** Flow cytometry analysis of NK cells shows significant downregulation of FOXO1 protein expression in miR-216a-3p transfected NK cells compared to control NK cells (n=2). **(B)** Dot plot representing ungated NK cells population. **(C)** Dot plot showing gated single NK cells population. **(D)** Histogram demonstrating unstained NK cells calibration **(E)** Histogram showing the protein expression of FOXO1 in control NK cells **(F)** Histogram representing the protein expression of FOXO1 in NK cells transfected with miR-216a-3p. **(G)** Overlay histogram showing the difference between FOXO1 protein expression in control NK cells (represented by blue color) and miR-216a-3p transfected NK cells (represented by red color). **(H)** Bar graph representing FOXO-1 protein levels in response to the introduction of *miR-216a-3p* mimics in NK cells. Results are expressed as mean +/- SEM. Asterisks indicate statistically significant difference, where *p<0.05.

Hence, we assessed the impact of *miR-216a-3p* mimicking on FOXO1 RNA and protein levels using qPCR and flow cytometry, respectively (**Figure 5**). Results showed that FOXO-1 was significantly downregulated on both the RNA level (n=3, 0.590, *p*-value=0.001, **Figure 5A**) and protein level (n=2, 86.67, *p*-value=0.005, **Figures 5B-H**) in response to the introduction of *miR-216a-3p* in NK cells compared to cells transfected with the negative control (**Figure 5**). Concerning the flowcytometry analysis of FOXO1, gating of single-cell NK population was performed according to the cell size (forward scatter) and granularity (side scatter). Ungated and single-cell NK cell populations are represented in **Figure 5B, C**, respectively. Unstained NK cells were used for calibration (**Figure 5D**). Demonstration of the protein level of FOXO-1 in control NK cells and NK cells transfected with *miR-216a-3p* mimics are represented in **Figures 5E, F**, respectively, and an overlay histogram demonstrated the difference between FOXO-1 protein expression in control NK cells and *miR-216a-3p* transfected NK cells (**Figure 5G**).

## 4 Discussion

MiRNAs have shown a pattern of opposing roles in immune cells compared to solid tumors, such as HCC. For example, many miRNAs have been characterized to play an oncogenic role in HCC, while maintaining a pro-cytotoxic role in NK cells of patients with HCC, thereby supporting tumor eradication. For instance, *miR-182* and *miR-615-5p* exhibit contradicting roles in NK cells versus HCC cell lines. *MiR-182* acts as an oncomiR in HCC cells through different targets, such as *ephrinA5* and *FOXO3a*, while it enhances the cytotoxicity of NK cells potentially through its effect on *NKG2D* (22, 24, 46). Conversely, *miR-615* has been reported to act as a tumor suppressor in HCC, while enhancing the anti-cytotoxic effect of NK cells, both achieved through the same target *IGF-1R* (23, 29). This highlights the possible tissue and pathway specific roles of miRNAs, and the need to study each of them in a tissue- and organ-specific context.

*MiR-216a* is a controversial miRNA composed of the two isomiRs, miR-216a-5p and miR-216a-3p. While extensive research has investigated the role of *miR-216a-5p* in many solid tumors and immunity, the role of *miR-216a-3p* remains vague, with limited knowledge of its action (30). There is no evidence on the expression levels of *miR-216a-3p* in NK cells or on whether it advocates a pro- or anti-cytotoxic impact for NK cells against tumors. It has been reported to act as a tumor suppressor in HCC, increasing sorafenib sensitivity through the downregulation of MAPK13 and modulating the MEK/ERK and ATF2 signaling pathways (37). This triggered our interest to investigate its function in NK cells, thereby illustating the multifaceted role of miR-216a-3p to investigate its potential utilization as a therapeutic target in cancer therapy. Therefore, this work aimed to examine the role of *miR-216a-3p* in the regulation of NK cell activity and function, while determining the expression status of this miRNA in NK cells of patients with HCC.

To achieve our aim, we isolated NK cells from whole blood of patients with HCC and healthy controls to assess *miR-216a-3p* expression profile. Furthermore, the levels of *miR-216a-3p* were manipulated in NK cells isolated from healthy controls to replicate their status in NK cells of HCC patients. This was followed by a series of functional analysis experiments to determine the impact of this manipulation on NK cells in terms of effector molecules release and cytolytic activity. Finally, we explored a possible mechanistic regulation imposed by *miR-216a-3p* on NK cells through studying one of its potential functional targets.

Upon screening *miR-216a-3p*, the relative expression levels were found to be significantly elevated approximately four-fold in NK cells of patients with HCC compared to healthy controls (**Figure 1**). To the best of our knowledge, the expression level of *miR-216a-3p* has never been investigated in any immune cells. However, a similar pattern of expression was observed in oral and gastric cancers, while it was found to be downregulated in colorectal, cervical, lung and breast cancers.

In these cancers, *miR-216a-3p* showed opposing roles. On one hand, it was found to act as an oncomiR in oral cancer through affecting the Wnt3a/β−catenin pathway (47). On the other hand, *miR-216a-3p* has been shown to act as a tumor suppressor regulating cell viability, proliferation and migration through various pathways in several types of cancer, such as in HCC, cervical, lung, colorectal, gastric and breast cancers (35, 37, 47–49). These data highlight the variable role of *miR-216a-3p*, which further intrigued our curiosity to determine its mechanistic role in NK cells. Thus, we followed up with a series of functional analysis experiments in which NK cells isolated from healthy controls were subjected to an ectopic expression of *miR-216a-3p* to mimic their overexpressed levels observed in NK cells of patients with HCC.

Ectopic expression of *miR-216a-3p* in NK cells isolated from controls caused decreased release of both TNF-ɑ and IFN-gamma concentrations (**Figure 2**). The decrease in TNF-ɑ concentration was nearly 75% (**Figure 2A**), while IFN-gamma release decreased by almost 50% (**Figure 2B**). There is no empirical data reported in literature regarding the impact of *miR-216a-3p* on TNF-ɑ and IFN-gamma production from NK cells or any other immune cells, punctuating the novelty of this finding. A multi-omics analysis study in colon adenocarcinoma predicted that high levels of *miR-216a-3p* in the cancer cells may have a negative immunomodulatory effect, accompanied by a decrease in NK-like T-cells, leading to decreased overall survival (36). In contrast, a study by Song et al. showed that the overexpression of *miR-216a-3p* during bone marrow mesenchymal stem cell differentiation into type II alveolar epithelial cells caused an elevation in the release of the pro-inflammatory molecules TNF-ɑ and IFN-gamma (49). This contradiction might be due to the different cell types being investigated, once again highlighting the tissue-specific effects of miRNAs and the necessity to study their roles within different cellular contexts.

We then proceeded to assess the impact of *miR-216a-3p* on NK cell activity by determining the levels of GrB (**Figure 2C**) and PRF (**Figure 2D**), which are produced by NK cells to mediate cytolysis of target cells, including tumor cells (50). Our results showed that both GrB and PRF levels decreased by approximately 40% and 30%, respectively (**Figures 2C, D**, respectively). To our knowledge, there is no other evidence in literature that correlated either *miR-216a-3p* expression with the levels of these effector molecules as an indicator of NK cell activity. Other miRNAs however, have shown similar patterns on these effector molecules; for example, *miR-615-5p* reduced perforins in NK cells (23), and *miR-519a-3p* showed similar behavior in reducing both PRF and GrB in breast cancer cells (51). These data and our findings highlight the ability of miRNAs to regulate effector molecule release.

Since the overexpression of *miR-216a-3p* led to a significant decrease in effector molecules, we were interested in investigating whether this would be reflected on the overall cytolytic activity of NK cells upon co-culturing with HCC cells. Therefore, as a final step towards assessing the role of *miR-216a-3p* in regulating NK cell activity, we performed the LDH cytotoxicity assay, where elevated LDH enzyme activity indicates the cytolytic activity of NK cells mediated through its effector molecules. The LDH assay has indicated a significant decrease in the cytotoxicity of NK cells transfected with *miR-216a-3p* against Huh-7 cells (**Figure 3**). A similar pattern was observed with *miR-615-5p*, as it was shown to suppress NK cytolytic activity against Huh-7 cells through the suppression of *NKG2D*, an NK activating receptor (23, 28, 29).

Finally, we carried out an *in silico* analysis accompanied by a literature review to identify a potential *miR-216a-3p* downstream target, through which its anti-cytotoxic effect in NK cells may be mediated (**Figure 4**). KEGG analysis of *miR-216a-3p* predicted targets showed significant enrichment in the FOXO-signaling pathway. Within this pathway, *FOXO-1* an*d FOXO-4* were two FoxO family members which were identified through miRDB as predicted *miR-216a-3p* target genes. *FOXO*s are widely considered tumor suppressor transcription factors. The FOXO family consists of four members: *FOXO-1*, *3*, *4* and *6* (52). *FOXO-1*, *3*, and *4* are ubiquitously expressed, while *FOXO-6* expression is restricted to the central nervous system (52). The protein abundance of FOXO-1 and FOXO-4 was determined through the *Human Protein Atlas*, which showed a very low and nonspecific expression of FOXO-4 in PBMCs, including NK cells and other lineages of immune cells. On the other hand, *FOXO-1* has the highest expression in immune B- and T-cells and is the only member of the family that was previously investigated in NK regulation, where it modulates NK maturation, development, and cytotoxicity (43, 52, 53). FOXO-1 has been shown to orchestrate the induction of autophagy as it interacts with Atg7, and its deficiency negatively impacts the development of NK cells (43). Nevertheless, the mechanism by which FOXO-1 affects NK cell cytotoxicity remains controversial and unclear. On one hand, FOXO-1 has recently been described to negatively impact NK maturation and cytotoxicity (54). On the other hand, it was shown in a different study to be crucial for NK cell development and clearance of murine cytomegalovirus (43). Hence, we assessed the impact of *miR-216a-3p* on FOXO-1 expression in NK cells. Our results showed that upon the overexpression of *miR-216a-3p* in NK cells, FOXO-1 mRNA and protein levels were both significantly decreased (**Figures 5A, H**), indicating the miR-216a-3p might induce its functional impacts in NK-cells partially through regulating FOXO1.

Overall, our work offers a potential molecular basis through which solid malignancies can evade immune action, one of the hallmarks of cancer, via the upregulation of *miR-216a-3p* in NK cells of patients with HCC. Our results point to the potential use of *miR-216a-3p* inhibitors to restore the activity of NK cells in patients with HCC. Recent studies have explored the use of different nanoformulations designed to optimize the targeted delivery of miRNAs, particularly *miR-34*, thereby enhancing their anticancer therapeutic efficiency (27). Similarly, further studies could be performed to enhance the targeting precision of *miR-216a-3p* inhibitors into NK cells and explore their anticancer potential in HCC and other solid malignancies.

**Figure 6:**
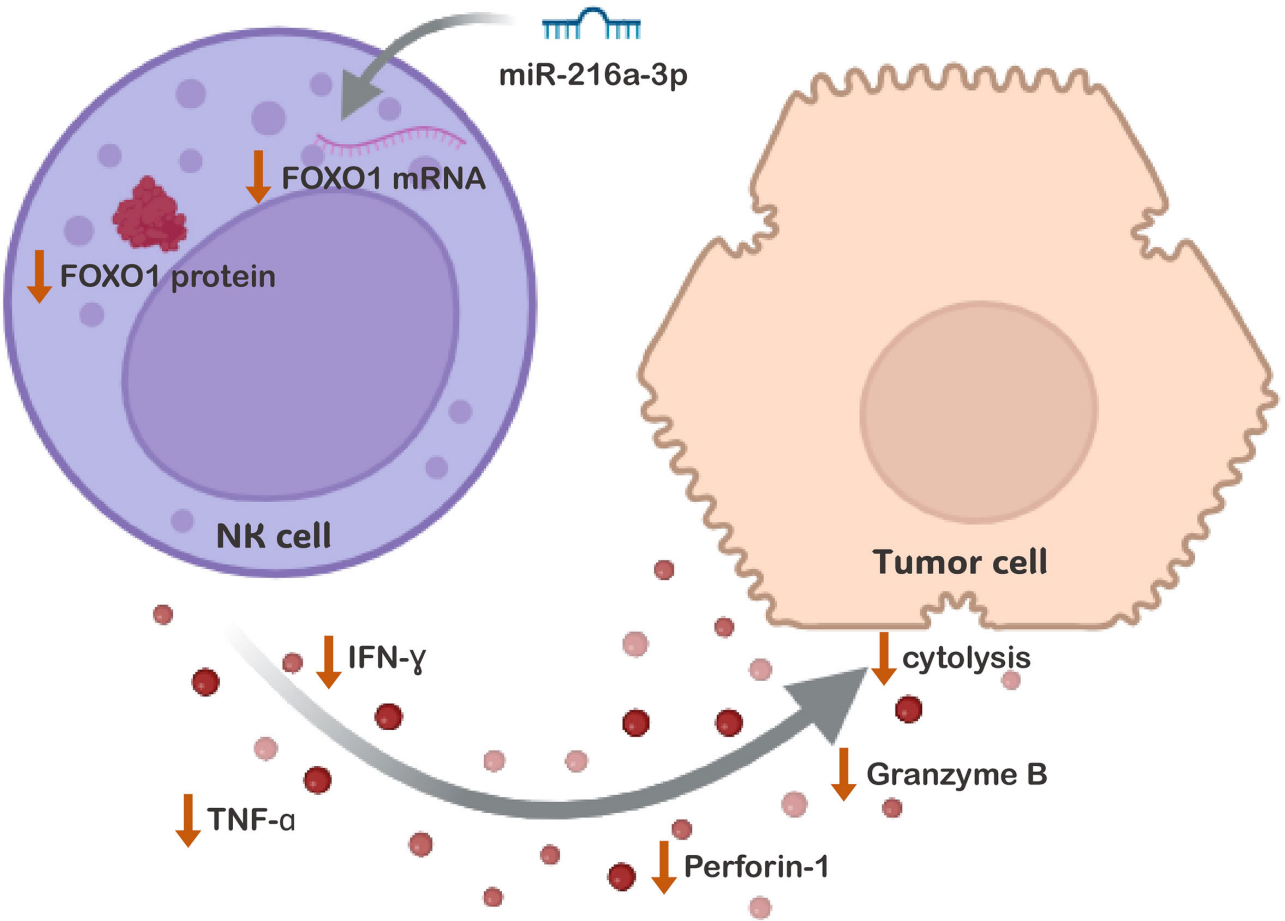
The Impact of *miR-216a-3p* NK Cells Anti-tumor Activity and Cytolytic Function. The proposed mechanistic pathway through which ectopic levels of *miR-216a-3p* affects the anti-tumor and cytolytic activity of NK cells against its target cells. The activity of NK cells was shown to be impacted on many levels, represented by the decrease in the release of cytokines and effector molecules. This was accompanied by a decline in LDH cytolytic activity. This might have been achieved through the downregulation of FOXO-1, identified as one of *miR-216a-3p* targets among the enriched FoxO Signaling pathway.

In conclusion, our results have shown that *miR-216a-3p* was upregulated in NK cells of patients with HCC and that it plays an anti-cytotoxic role in NK cells (**Figure 6**). Furthermore, *miR-216a*-3p suppressed the expression of FOXO-1, suggesting that *FOXO1* might be one of the targets through which *miR-216a-3p* imposes its anti-cytotoxic effect in NKs (**Figure 6**).

## Data Availability

The original contributions presented in the study are included in the article/**Supplementary Material**. Further inquiries can be directed to the corresponding author.
